# Complex posterolateral rotatory instability due to malunited radius fracture: diagnosis with four-dimensional computed tomography scan and corrective osteotomy

**DOI:** 10.1016/j.xrrt.2026.100840

**Published:** 2026-08-05

**Authors:** Anton Ferdinand Schmidt, Doruk Akgun, Stephan Pauly, Sven Märdian, Katja Ruettershoff, Jan-Philipp Imiolczyk, Kathi Thiele

**Affiliations:** a Medical University of Vienna, Vienna, Austria; b Clinic for Shoulder Surgery, Vivantes Auguste-Viktoria-Klinikum, Berlin, Germany; c Charité - Universitätsmedizin Berlin, Center for Musculoskeletal Surgery, Berlin, Germany; d Department of Trauma, Hand and Reconstructive Surgery, Rostock University Medical Center, Rostock, Germany; e University Hospital Basel, Basel, Switzerland; f Department for Shoulder and Elbow Surgery, Schulthess Clinic, Zurich, Switzerland

## Introduction

Fractures of the radial head are the most common type of fracture around the elbow joint with an incidence of 55 per 100,000.^3^ Although they can occur as an isolated injury, they are often accompanied by additional fractures, ligament injuries, and dislocation.^9^ In some cases, these injuries may result in posterolateral rotatory instability (PLRI).^12^ Although ligamentous insufficiency is a key component in the pathogenesis of PLRI, osseous integrity also plays a critical role. In chronic cases, this may involve osseous defects of the posterolateral aspect of the elbow, such as the Osborne-Cotterill lesion or delayed PLRI secondary to cubitus varus deformity.^8^^,^^14^ Post-traumatic deformities leading to PLRI are rare and scarcely reported in the literature. The radial head plays a crucial role in joint stability and load transmission. It acts as a stabilizer against valgus stress and provides longitudinal stability of the forearm. The unique anatomic orientation of the radial head with a radial head shaft angle of about 163-168° and an elliptical form significantly contributes to elbow stability: in supination, the extension lies posteriorly to the capitulum, counteracting biceps-induced anterior translational forces while lifting objects. In pronation this lip shifts anteriorly, forming an additional barrier against posterior dislocation such as those occurring during falls.^2^^,^^10^^,^^11^^,^^16^ The restoration of the radiocapitellar articulation is a key consideration when deciding on surgical intervention.^5^^,^^7^^,^^13^ Hackl et al^5^ demonstrated that instability is the second most common reason for revision surgery following radial head fractures, with 38.2% of cases presenting with PLRI stages II–III. Post-traumatic malformations causing PLRI are also known to occur in cases of supracondylar humeral fractures. Varus malalignment and the medializing pull of the triceps set biomechanical alterations in motion, including external rotation of the ulnar causing elongation of the lateral collateral ligament (LCL) and resulting in PLRI.^8^ Eckl et al^4^ described an elliptical malunion following complex radial head fractures (Mason type III), which led to the elongation of the lateral ligamentous complex. This deformity, caused by decentration of the radial head through the so-called cam effect, led to PLRI. In the case of Eckl et al^4^, multiple revision procedures were required. Ultimately, resection of the osseous deformity combined with ligament reconstruction achieved a satisfactory and stable outcome.

This case report is about a complex presentation of PLRI caused by a post-traumatic deformity of the radial neck, resulting from a malunited fracture sustained 2 years earlier. In addition to clinical examination and plain radiographs, dynamic four-dimensional computed tomography (4D-CT) helped confirm the diagnosis by visualizing both the functional instability pattern and the underlying post-traumatic malalignment of the proximal radius. The patient was informed, and consent was obtained to publish details about the case, including all accompanying material.

### Case presentation

A 16-year-old female patient presented to our outpatient clinic with persistent lateral elbow pain and limited range of motion of the right elbow ongoing since a traumatic elbow dislocation with a conservatively treated radial neck fracture sustained during trampoline jumping 2 years before. The injury had initially been managed by closed reduction and conservative treatment in another medical center. Despite consistent physiotherapy, the patient reported a persistent position- and exertion-dependent pain with motion deficits. On clinical examination, there was tenderness over the radial head and lateral epicondyle. Active elbow motion showed an extension deficit of 30° and flexion to 150°. Forearm rotation was 90° of pronation and 80° of supination. The tabletop relocation test was positive, whereas the pincer grip test was negative, providing only partially consistent clinical evidence of PLRI. Gross and fine motor functions of the hand were intact, and no sensory deficits were noted in the elbow, forearm, or hand. Conventional radiographs suggested a bony deformity with posterolateral displacement of the radial head caused by malalignment of the radial shaft at the level of the radial tuberosity. Magnetic resonance imaging (MRI) evaluation showed no evidence of ligamentous injury. However, based on static radiographs and clinical examination alone, the deformity and its functional relevance could not be assessed with sufficient certainty to proceed to surgical correction. Therefore, 4D-CT was performed for further 3-dimensional and functional assessment in this case.

The 4D-CT examination was performed as a stationary dynamic volume CT acquisition of the elbow on a 320-detector-row CT scanner (Aquilion ONE, Toshiba/Canon Medical Systems, Ōtawara, Tochigi, Japan). Relevant acquisition parameters were tube voltage 100 kV, tube current 50 mA, rotation time 0.275 s, exposure 13 mAs per rotation, slice thickness 0.5 mm, small reconstruction field of view, and iterative reconstruction using adaptive iterative dose reduction three dimension (3D) standard. The dose-length product was 52.8 mGy cm, corresponding to an estimated effective dose of approximately 0.04 mSv using an extremity conversion coefficient of 0.0008 mSv/(mGy·cm).

**Figure 1:**
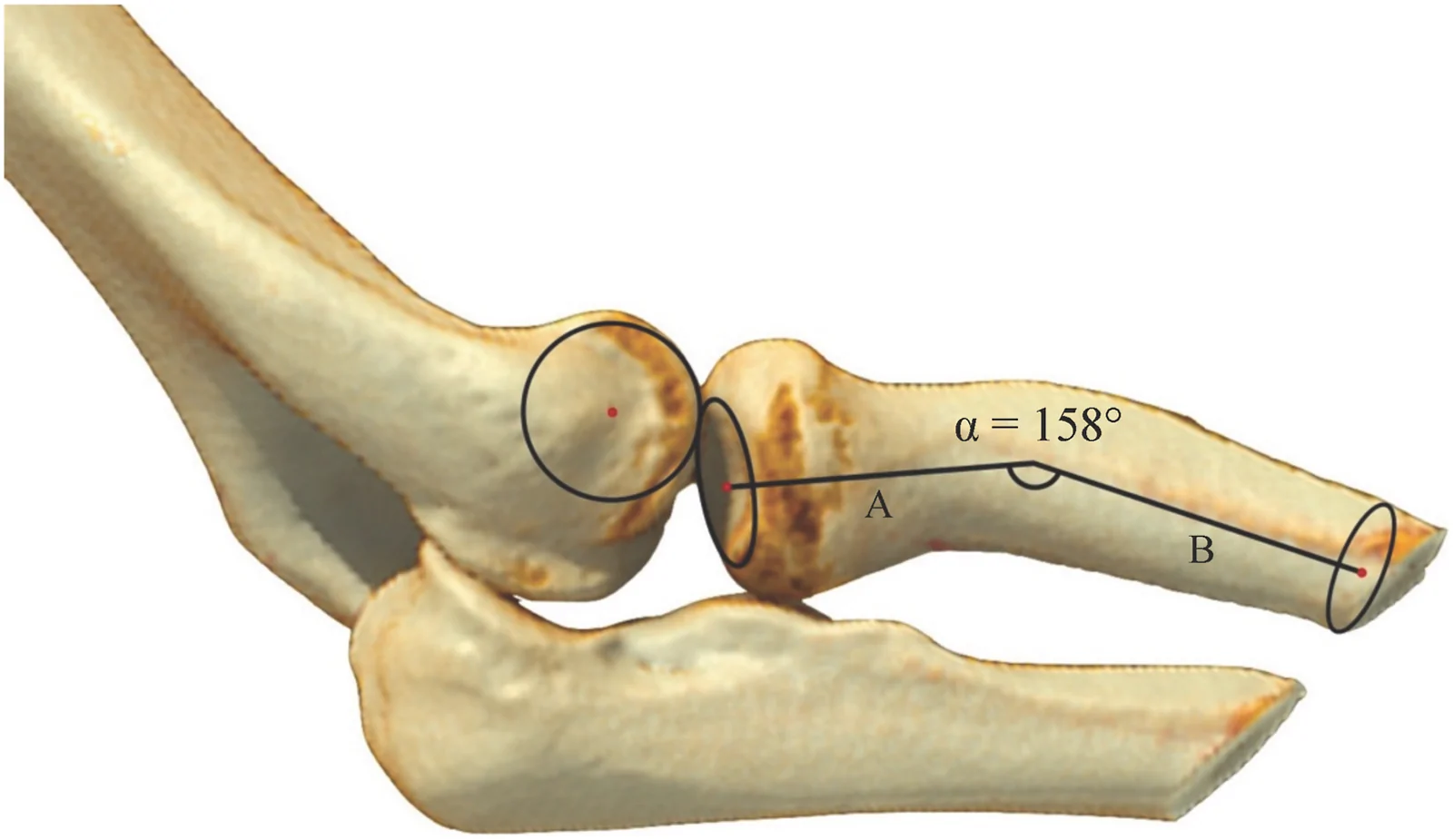
Computed tomography in full supination showing the bony deformity: radial head axis (A), shaft/diaphysis axis (B), radial head shaft angle (α).

This provided crucial insight into the underlying mechanism of the persistent subluxation (Video 1). The dynamic CT demonstrated a reproducible posterolateral subluxation of the ulnoradial unit in full supination, with an approximate 158° radial head shaft angle showing a posteromedial tilt of the radial head's articular fovea and subluxation (Fig. 1). Based on these findings, we concluded that a post-traumatic malunion of a proximal radial fracture had led to a levering cam effect. The malalignment appeared to alter joint kinematics such that, during forearm supination, abnormal radiocapitellar contact forces contributed to posterolateral translation of the radial head. Although the lateral ligamentous structures appeared intact on MRI, a subtle functional attenuation could not be excluded preoperatively. Therefore, the patient was counseled about the possibility of additional ligament reconstruction, either as an intraoperative option or as a staged secondary procedure if recurrent instability remained post-operatively.

### Surgical procedure

**Figure 2:**
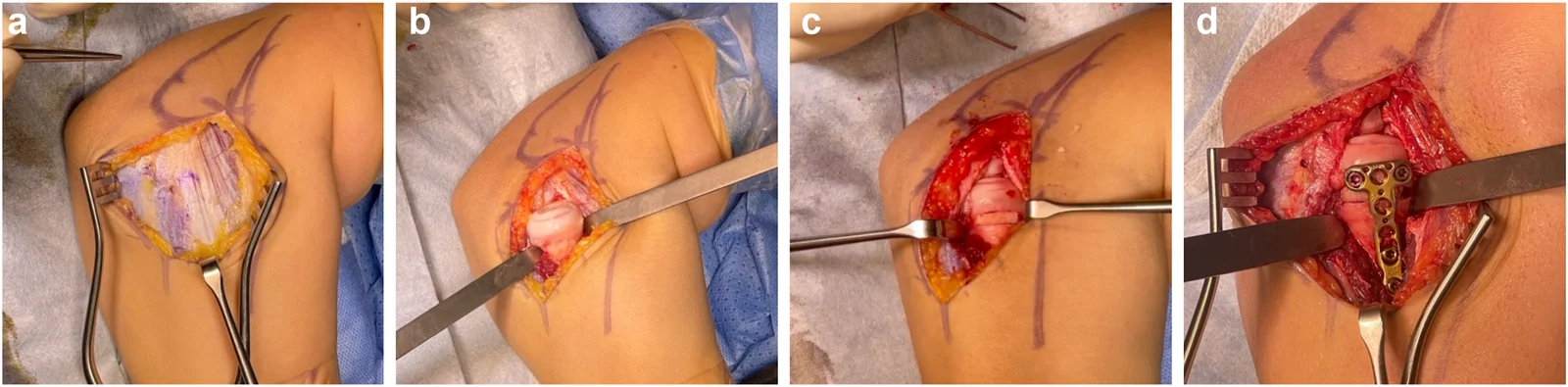
Intraoperative technique: lateral approach (**a**), visualization of the radial head deformity (**b**), open-wedge osteotomy with bone allograft (**c**), osteosynthesis (**d**).

**Figure 3:**
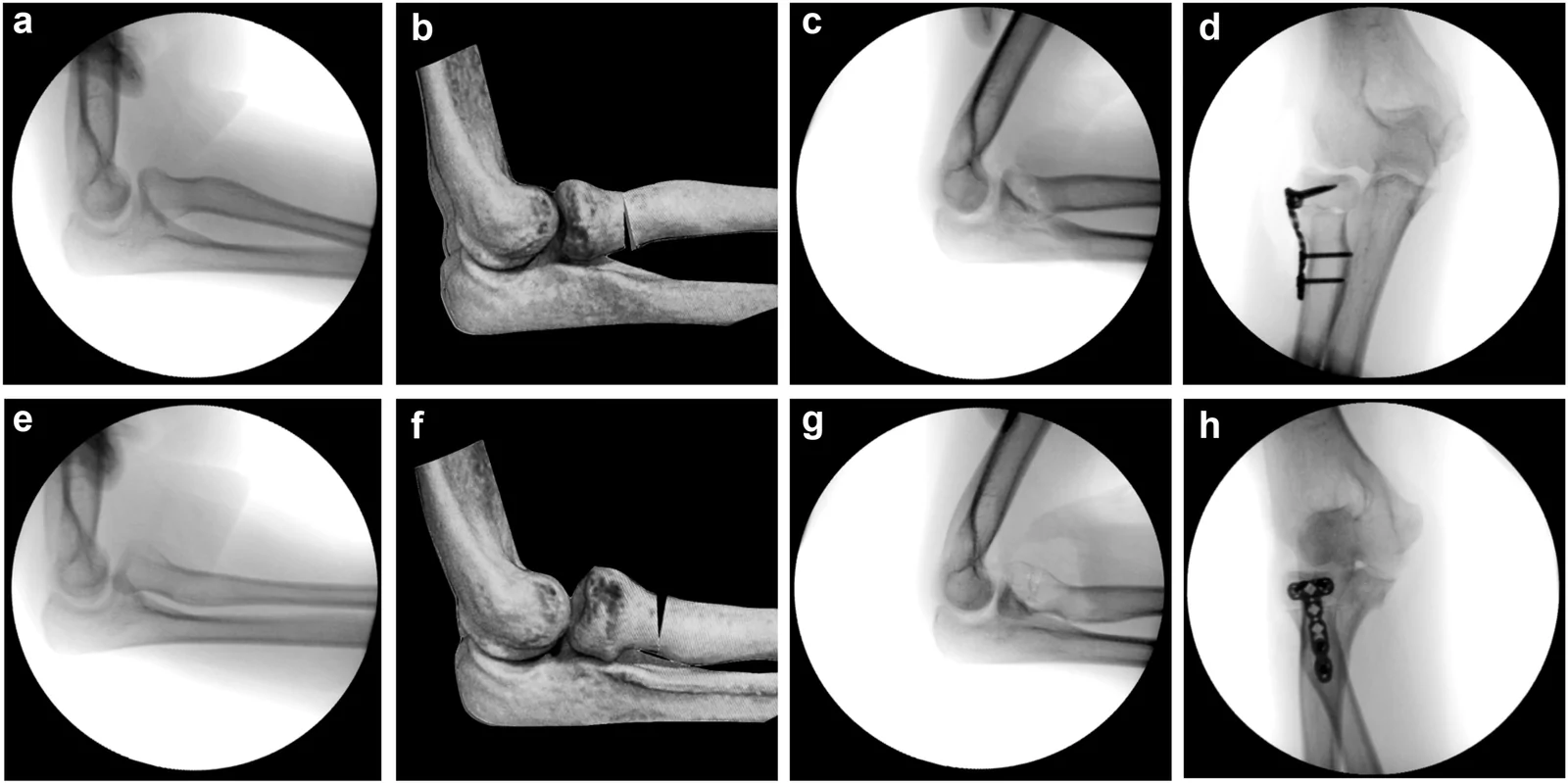
Intraoperative technique: native axis in supination (**a**) and pronation (**e**), planning of the open-wedge osteotomy (**b, f**), osteotomy with bone allograft (**c, g**), centered joint after T-plate osteosynthesis (**d, h**).

The procedure was performed under general anesthesia with the patient placed in a supine position. The right arm was positioned in pronation on a side table. A lateral approach was chosen, entering just anterior to the Kocher muscle interval in line with an extensor digitorum access (extensor digitorum communis approach) to minimize the risk of nerve or ligament injury (Fig. 2*A*). The exposure was performed in pronation for the same reason. After careful preparation and exposure of the proximal radius, the area of deformity was visualized (Fig. 2*B*). The lateral ligamentous structures appeared macroscopically intact. Intraoperative fluoroscopy confirmed the most pronounced axis deviation in supination (Fig. 3*A*). The arm was then brought into pronation under fluoroscopic guidance, and the planned osteotomy was performed (Fig. 2, *C* and *D*, Fig. 3, *C* and *G*). The correction was performed under fluoroscopic control by cautious, gradual widening of the osteotomy site with chisels in the manner of an open-wedge osteotomy until centralization of the radial head was achieved. A structural bone allograft wedge was inserted into the osteotomy site, resulting in significant correction of radial head alignment and immediate improvement in rotational axis congruity (Video 1). Subsequent hypersupination testing throughout the full range of motion showed no relevant joint decentration, and ligament reconstruction was therefore not performed.

For additional stabilization, a 2.4 mm T-plate (FA. Synthes, Switzerland) was applied and secured with screw fixation. Intraoperative fluoroscopy confirmed satisfactory alignment and hardware positioning. Passive intraoperative elbow motion showed a 10° extension deficit with flexion to 150°. Forearm rotation was 90° of pronation and 80° of supination. The muscle interval and antebrachial fascia were adapted, followed by the closing of subcutaneous tissue and skin in standard fashion. A dorsal splint for initial post-operative immobilization was placed.

### Post-operative procedure and outcome

Post-operative immobilization was initiated with a dorsal plaster splint for 7 days, followed by using an articulated elbow brace for 5 weeks. Varus and valgus loading were avoided for a total of 4 months to protect the osteotomy and fixation. Passive mobilization was initiated on the first post-operative day with the forearm in pronation. During the first 3 weeks, elbow motion was limited to 20° of extension and 90° of flexion. For the following 2 weeks, passive flexion was increased to 110° while maintaining the 20° extension limit. A gradual transition to active range of motion exercises was initiated 7 weeks post-operatively.

**Figure 4:**
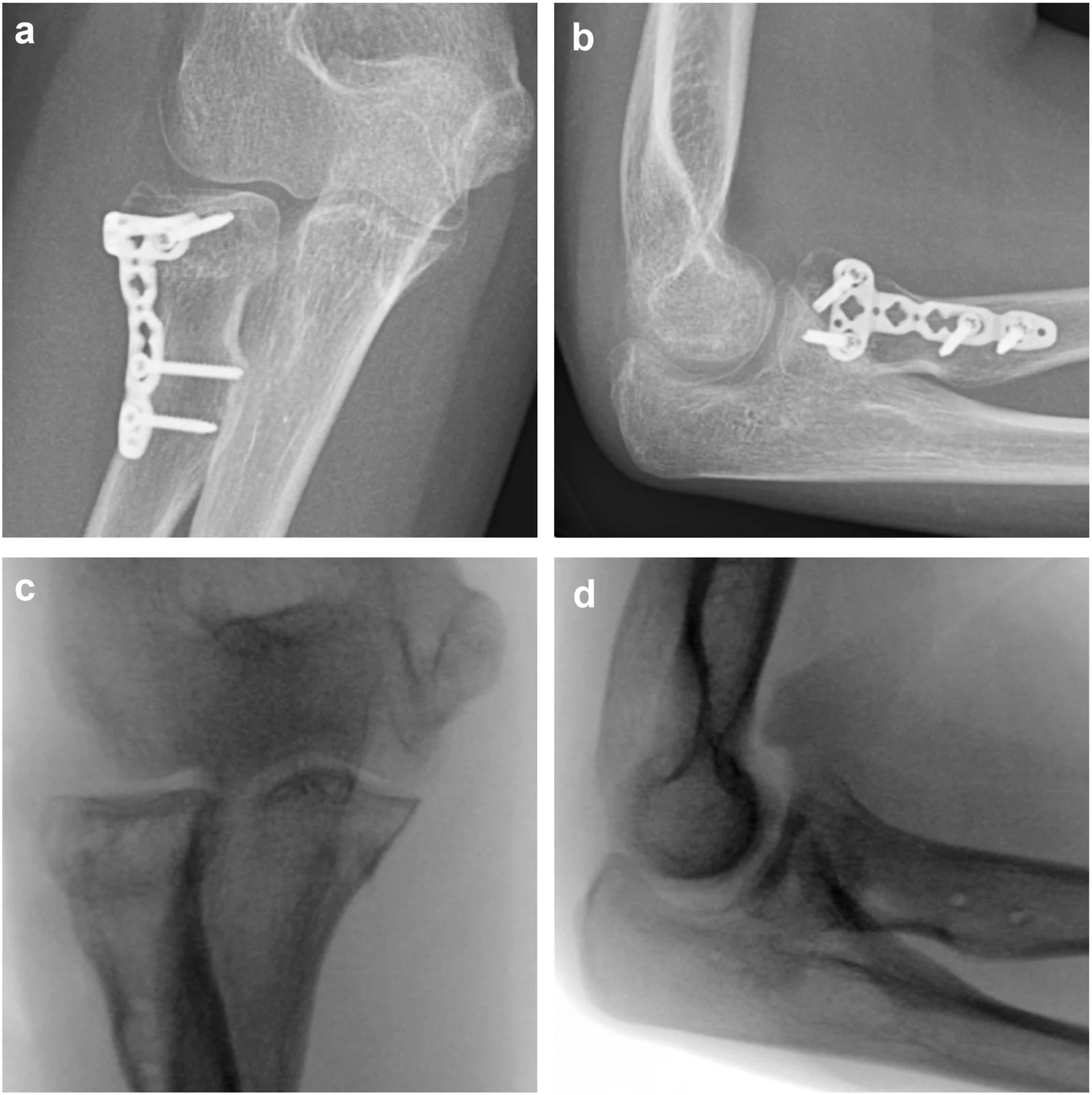
Follow-up radiographs: radiographs 3 months post-operatively (**a, b**) and radiographs after plate removal (**c, d**).

At the 3-month follow-up, the patient was pain free and reported marked improvement of her pre-operative symptoms, including full resolution of instability complaints, allowing unrestricted performance of daily activities with a slight extension deficit. Elbow motion showed a 20° extension deficit with flexion to 150°. Forearm rotation was 70° of pronation and 70° of supination. Radiological evaluation demonstrated a well-centered elbow joint, complete incorporation of the allograft, and unchanged correct positioning of the osteosynthesis (Fig. 4, *A* and *B*). The fixation plate was subsequently removed 15 months after the procedure in accordance with the patient's preference (Fig. 4, *C* and *D*).

**Figure 5:**
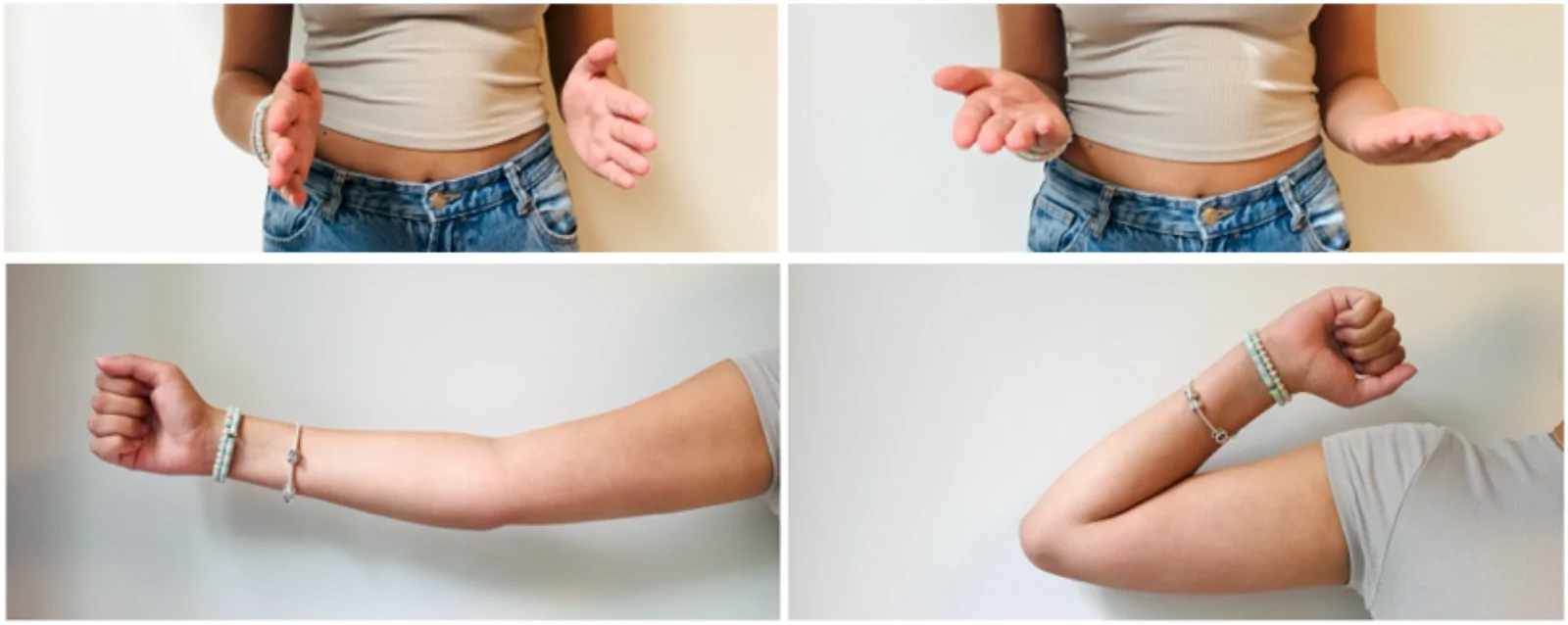
Range of motion at 18-month follow-up.

Eighteen months after surgery, the patient continued to be asymptomatic, presenting with a normal radiographic appearance and no signs of pain, apprehension, or joint subluxation and only a slight extension deficit. The tabletop relocation and pincer-grip tests were negative. Elbow motion showed a 20° extension deficit with flexion to 150°. Forearm rotation remained good, with 80° of pronation and 80° of supination, corresponding to a minimal persistent extension deficit and slight limitation of terminal supination (Fig. 5).

At a 45-month follow-up, clinical and functional outcomes remained good. Stability testing under valgus and varus stress and the push-up test demonstrated no signs of instability or pain. Active motion further improved to a 10° extension deficit and 150° of flexion. Forearm rotation remained 80° of pronation and 85° of supination, with no pain at rest (Visual Analog Scale, 0/10) and slight pain under loading (Visual Analog Scale, 2/10). At final follow-up, the patient-reported outcome was good, with a Quick Disabilities of the Arm, Shoulder and Hand Questionnaire (QuickDASH) score of 6.8 points, indicating minimal residual upper-extremity disability.

The patient reported no sport-specific limitations and no restrictions in activities of daily living.

## Discussion

4D-CT can play a crucial role in the diagnosis and understanding of cases with complex bony impingement.^1^ This was particularly emphasized by Seah et al^15^ for the elbow. They demonstrated that 4D-CT led to a change in diagnosis or management in over 50% of cases involving dynamic elbow disorders. They advocate its use in the elbow due to the limitations of conventional imaging, which often fails to detect subtle dynamic pathologies. 4D-CT enables real-time visualization of joint kinematics throughout the range of motion, making it particularly valuable in identifying motion-dependent impingement, instability, and subluxation.^15^ In the present case, conventional imaging demonstrated the proximal radial deformity, and clinical examination suggested PLRI. However, the dynamic relationship between the deformity and the radiocapitellar joint during forearm rotation remained unclear.

The 4D-CT examination provided relevant additional information by combining precise assessment of the proximal radial head-axis deformity and dynamic visualization of reproducible posterolateral subluxation. This supported the interpretation that the instability was related to the osseous deformity rather than an isolated ligamentous lesion.

Radiation exposure is an important consideration when using CT scans, particularly in pediatric and adolescent patients. The acquisition was restricted to the clinically relevant elbow region and performed using a low-dose protocol with reduced tube voltage/current, a small reconstruction field of view, and iterative reconstruction. The estimated effective dose was approximately 0.04 mSv, which is well below the reported average effective dose of conventional elbow CT (0.21 ± 0.11 mSv).^6^ Nevertheless, 4D-CT should not be regarded as a routine diagnostic tool in adolescent patients with elbow instability. Its use should be reserved for carefully selected cases in which conventional imaging and clinical or fluoroscopic assessment do not sufficiently explain the dynamic pathology and where the expected diagnostic gain may directly influence treatment planning.

Although the LCL complex appeared intact on pre-operative MRI and was also found to be macroscopically intact intraoperatively, a functional attenuation or elongation of the LCL cannot be completely excluded. Given the long-standing, recurrent posterolateral subluxation, chronic stretching of the lateral stabilizers may have contributed to the instability despite the absence of a discrete ligament rupture. Therefore, the favorable post-operative outcome may not solely reflect correction of the osseous deformity but may also have resulted from restoration of the bony alignment with secondary retensioning of the lateral ligamentous complex. Similar to the mechanism described by Eckl et al^4^, recurrent subluxation may eventually lead to ligamentous elongation. Therefore, a subtle functional insufficiency of the LCL complex cannot be definitively ruled out in the present case. The possibility of additional ligament reconstruction was discussed with the patient pre-operatively, including both an immediate reconstruction if relevant residual LCL insufficiency became evident intraoperatively and a staged secondary reconstruction in case of persistent or recurrent instability after bony correction. After osteotomy hypersupination testing throughout the full range of motion showed no relevant joint decentration. Ligament reconstruction was therefore not performed and has not been considered necessary given the stable post-operative course.

Although radial overstuffing was considered a potential concern after open-wedge osteotomy, clinically relevant radial overlengthening was considered unlikely in the present case because the initial fracture deformity was associated with post-traumatic shortening and impaction. The osteotomy was planned manually using conventional radiographs and 4D-CT findings, and the correction was performed freehand under fluoroscopic control with gradual widening of the osteotomy.

Digital 3D planning software, patient-specific instrumentation, and cutting guides were not available at our institution and were therefore not used. For this monoplanar correction without a relevant rotational component, planning based on conventional radiographs and 4D-CT findings was considered sufficient. Nevertheless, the absence of quantitative intraoperative assessment of radial length and patient-specific guidance must be acknowledged as a limitation. Digital 3D planning and patient-specific guidance may represent valuable alternatives for more precise correction, particularly in rotational deformities or more complex multiplanar cases, and may also help to better control radial length and reduce the risk of overstuffing.

## Conclusion

These findings highlight the potential role of osseous integrity in maintaining stability in the elbow joint. Disruption of the anatomical congruity, whether due to fracture, malunion, or deformity, can compromise the biomechanical balance of the elbow. In this 16-year-old patient, 4D-CT demonstrated reproducible posterolateral subluxation caused by a malunited proximal radial deformity. Corrective open-wedge osteotomy with structural bone allograft restored radial head alignment, eliminated dynamic subluxation, and achieved stable elbow function without the need for ligament reconstruction. At 45 months, the patient remained clinically stable with minimal residual disability and no relevant pain or activity-related limitations. In selected complex or atypical cases, 4D-CT may therefore provide valuable diagnostic and surgical planning information. However, its use must be carefully balanced against radiation exposure and should be reserved for cases in which relevant additional information for treatment planning can reasonably be expected, especially in adolescent patients.

## Supplementary Data

Video 1 4D-CT and intraoperative imaging demonstrating posterolateral rotatory instability and surgical technique.

## References

[1] Bell S.N., Troupis J.M., Miller D., Alta T.D., Coghlan J.A., Wijeratna M.D. (2015). Four-dimensional computed tomography scans facilitate preoperative planning in snapping scapula syndrome. J Shoulder Elbow Surg.

[2] Captier G., Canovas F., Mercier N., Thomas E., Bonnel F. (2002). Biometry of the radial head: biomechanical implications in pronation and supination. Surg Radiologic Anat.

[3] Duckworth A.D., Clement N.D., Jenkins P.J., Aitken S.A., Court-Brown C.M., McQueen M.M. (2012). The epidemiology of radial head and neck fractures. J Hand Surg.

[4] Eckl L., Braun C., Schoch C., Geyer S. (2025). Radial head deformity as an osseous cause of recurrent posterolateral elbow instability—a case report. Obere Extremität.

[5] Hackl M., Wegmann K., Hollinger B., El-Zayat B.F., Seybold D., Gühring T. (2019). Surgical revision of radial head fractures: a multicenter retrospective analysis of 466 cases. J Shoulder Elbow Surg.

[6] Iordache S.D., Goldberg N., Paz L., Peylan J., Hur R.B., Steinmetz A. (2017). Radiation exposure from computed tomography of the upper limbs. Acta Orthop Belg.

[7] Kupperman E.S., Kupperman A.I., Mitchell S.A. (2018). Treatment of radial head fractures and need for revision procedures at 1 and 2 years. The J Hand Surg.

[8] O’Driscoll S.W., Spinner R.J., McKee M.D., Kibler W.B., Hastings H., Morrey B.F. (2001). Tardy posterolateral rotatory instability of the elbow due to cubitus varus. J Bone Joint Surg Am.

[9] Pike J.M., Athwal G.S., Faber K.J., King G.J.W. (2009). Radial head fractures—an update. J Hand Surg.

[10] Porcellini G., Rotini R., Stignani Kantar S., Di Giacomo S. (2018).

[11] Pretorius H.S., Ferreira N., Burger M.C. (2021). A computer tomography-based anthropomorphic study of forearm osteology: implications for prosthetic design. SA Orthop J.

[12] Rhyou I.H., Lee J.-H., Kim K.C., Ahn K.B., Moon S.C., Kim H.J. (2017). What injury mechanism and patterns of ligament status are associated with isolated coronoid, isolated radial head, and combined fractures?. Clin Orthop Relat Res.

[13] Rineer C.A., Guitton T.G., Ring D. (2010). Radial head fractures: loss of cortical contact is associated with concomitant fracture or dislocation. J Shoulder Elbow Surg.

[14] Rotman D., Bokhari N., Wright A., Watts A.C. (2024). The posterolateral ligament of the elbow: anatomy and clinical relevance. J Shoulder Elbow Surg.

[15] Seah R.B., Mak W.-K., Bryant K., Korlaet M., Dwyer A., Bain G.I. (2022). Four-dimensional computed tomography scan for dynamic elbow disorders: recommendations for clinical utility. JSES Int.

[16] Van Riet R.P., Van Glabbeek F., Neale P.G., Bimmel R., Bortier H., Morrey B.F. (2004). Anatomical considerations of the radius. Clin Anat.

